# Combined Inflammatory Burden Index and hand grip strength for prognostic assessment in gastric cancer patients: a multicenter cohort study

**DOI:** 10.3389/fnut.2026.1906113

**Published:** 2026-09-09

**Authors:** Fuquan Yang, Xinyi Wang, Lishuang Wei, Haitao Wen, Pengyu Chen, Junfei Chen, Yuhang Zhang, Nuo Xu, Yibo Chen, Hanping Shi, Hailun Xie

**Affiliations:** Department of Gastrointestinal and Gland Surgery, The First Affiliated Hospital, Guangxi Medical University, Nanning, Guangxi, China

**Keywords:** gastric cancer, inflammatory burden index, handgrip strength, overall survival, malnutrition, prognosis

## Abstract

**Background:**

Gastric cancer (GC) is associated with heterogeneous prognosis, and individual inflammatory or nutritional markers have limited predictive value. This study aimed to develop and evaluate a novel grading system integrating the Inflammatory Burden Index (IBI) and handgrip strength (HGS) for prognostic assessment in GC.

**Methods:**

Kaplan–Meier curves and log-rank tests were used for survival comparisons. Multivariate Cox and logistic regression analyses were performed to evaluate the independent associations between the IBI-HGS grade and overall survival (OS) as well as severe malnutrition. Sensitivity analyses excluding early deaths were conducted to assess the robustness of the results.

**Results:**

This multicenter retrospective cohort study included 1,052 GC patients. During a median follow-up duration of 20.75 months, 455 deaths occurred. A three-tier IBI-HGS grade (low-, medium-, and high-risk) was established by combining IBI and HGS. Kaplan–Meier analysis demonstrated significant differences in OS among the IBI-HGS grades: 67.9% in the low-risk group, 58.4% in the medium-risk group, and 44.9% in the high-risk group (log-rank *p* < 0.001). Multivariate Cox regression analysis showed that the medium-risk grade was associated with a 69.1% higher risk of mortality (HR = 1.691, 95% CIs: 1.323–2.162, *p* < 0.001), while the high-risk grade was associated with a 123.0% increased risk (HR = 2.230, 95% CI: 1.690–2.942, *p* < 0.001). Multivariate logistic regression analysis confirmed that the medium-risk grade was associated with a 56.2% higher risk of severe malnutrition (OR = 1.562, 95% CIs: 1.167–2.091, *p* = 0.003), whereas the high-risk grade was associated with a 205.8% increased risk (OR = 3.058, 95% CI: 2.110–4.431, *p* < 0.001). Sensitivity analyses further confirmed these independent prognostic associations.

**Conclusion:**

The novel IBI-HGS grade, integrating systemic inflammatory burden and skeletal muscle function, is a simple, noninvasive, and clinically practical biomarker. It was independently associated with OS and baseline severe malnutrition, enabling refined prognostic stratification and supporting the identification of GC patients with poor nutritional status.

**Clinical trial registration:**

http://www.chictr.org.cn/showproj.aspx?proj=33675, ChiCTR1800020329.

## Introduction

Gastric cancer (GC) remains a major global public health burden, ranking fifth in incidence and fourth in cancer-related mortality worldwide, with East Asia—particularly China—bearing the highest disease burden (1–4). Despite advances in surgical and multimodal therapies, prognosis among GC patients remains heterogeneous, even in patients with the same TNM stage. Anatomical staging primarily reflects tumor burden but does not account for systemic host factors, such as inflammation, muscle function, and nutritional reserve, which are important determinants of GC outcomes. Therefore, there is an urgent need to develop comprehensive and readily accessible prognostic tools that integrate these multidimensional host factors to improve risk stratification and guide personalized management.

Systemic inflammation and skeletal muscle function are two tightly interlocked drivers of GC progression and unfavorable clinical outcomes, forming a self-amplifying vicious cycle: sustained elevation of circulating pro-inflammatory cytokines accelerates skeletal muscle protein breakdown and sarcopenic muscle wasting, while loss of muscle mass further weakens anti-tumor immune competence and exacerbates systemic inflammatory activation. This bidirectional interplay means evaluating either inflammation or muscle function in isolation cannot fully capture a patient’s overall host fitness and disease risk (5–9). Inflammatory markers such as neutrophil-to-lymphocyte ratio (NLR) and C-reactive protein (CRP) have proven prognostic value for GC, yet single indicators fail to quantify total systemic inflammatory load (10). The Inflammatory Burden Index (IBI), a novel composite index calculated as CRP × (neutrophil/lymphocyte), has emerged as a potential inflammatory biomarker in recent studies (11–13). It provides a more comprehensive reflection of the cumulative systemic inflammatory response and demonstrates better prognostic performance than conventional individual inflammatory markers in cancer. Even so, IBI exclusively reflects inflammatory status and ignores skeletal muscle reserve, a core determinant of treatment tolerance and malnutrition risk. Handgrip strength (HGS), a simple and objective bedside measure of skeletal muscle mass and function, is a well-established standard for assessing nutritional status and sarcopenia in cancer patients (14–16). Reduced HGS is closely associated with malnutrition, impaired treatment tolerance, and poor survival in GC (17, 18). However, HGS alone does not reflect the systemic inflammatory burden that contributes to muscle wasting and disease progression.

To our knowledge, no previous study in GC has integrated the IBI, a comprehensive inflammatory biomarker, with HGS, a functional readout of skeletal muscle reserve, to construct an integrated prognostic grading system. Single-biomarker evaluations focusing solely on inflammation or muscle function fail to fully capture the reciprocal pathogenic axis linking systemic inflammation, sarcopenia, and malnutrition, leaving an unmet clinical demand for a combined stratification tool that accounts for both components of this vicious cycle. Joint incorporation of IBI and HGS addresses this critical limitation, as the composite metric simultaneously quantifies three interrelated drivers of GC progression: cumulative systemic inflammatory burden, skeletal muscle functional status, and nutritional reserve. By merging these mutually complementary dimensions, the IBI–HGS grading system delivers more holistic and precise prognostic discrimination than either biomarker used in isolation. Against this backdrop, the present multicenter cohort study sought to establish this novel IBI–HGS risk stratification scheme and validate its independent predictive performance for overall survival (OS) and severe malnutrition, aiming to furnish clinicians with a simple, evidence-based tool for individualized GC risk assessment.

## Materials and methods

### Study population

The patients included in this study were enrolled in the Investigation on Nutrition Status and its Clinical Outcome of Common Cancers (INSCOC) project, a multicenter study initiated in China in 2013. Eligible patients met the following inclusion criteria: (1) age 18–80 years; (2) histopathologically confirmed primary gastric adenocarcinoma; (3) complete baseline data on inflammatory indicators, HGS, and clinical characteristics; and (4) provision of written informed consent. The exclusion criteria were as follows: (1) concurrent multiple malignant tumors; (2) active systemic infection or acute inflammatory conditions; (3) severe comorbidities (e.g., heart failure, renal failure, or hepatic decompensation); and (4) incomplete follow-up data or missing key variables. In this study, a total of 1,052 patients from 27 participating centers were included. Patients were recruited between May 7, 2013, and December 31, 2020. The study protocol was approved by the Institutional Review Board of the First Affiliated Hospital of Guangxi Medical University and all participating centers and was registered in the Chinese Clinical Trial Registry (ChiCTR1800020329). All procedures were conducted in accordance with the Declaration of Helsinki.

### Data collection

Data were systematically collected from electronic medical records, standardized clinical assessment forms, and routine laboratory databases using unified protocols across all participating centers to ensure consistency. Demographic and clinical variables included age, sex, smoking and drinking history, family history of cancer, TNM stage (8th AJCC edition), antitumor treatments (surgery, radiotherapy, and chemotherapy), and comorbidities (hypertension and type 2 diabetes). Hypertension was defined as systolic blood pressure ≥140 mmHg and/or diastolic blood pressure ≥90 mmHg or regular use of antihypertensive medication, while type 2 diabetes was diagnosed according to WHO criteria. Fasting venous blood samples were collected within 72 h of hospital admission, prior to the initiation of any anti-tumor therapy including surgery, chemotherapy and radiotherapy, to exclude confounding alterations in inflammatory biomarkers induced by treatment. These specimens were analyzed for routine blood parameters (neutrophil and lymphocyte counts), C-reactive protein (CRP; immunoturbidimetric assay), and serum albumin (ALB; bromocresol green method), which were used to calculate the IBI as CRP × (neutrophil/lymphocyte). HGS was measured using a standardized dynamometer with 0.1 kg precision in the seated position. Participants performed maximal voluntary grip strength using the dominant hand, and two measurements were obtained. The maximum value was recorded for analysis. Sex-specific cutoff values were applied to define low HGS (<28 kg for males and <18 kg for females). All participating centers followed unified measurement procedures, including participant positioning, device operation, and data recording criteria, to minimize inter-center variability. All raw data were anonymized and independently verified by trained researchers to ensure accuracy.

### Follow-up and outcome

All enrolled patients were prospectively followed from the date of baseline assessment completion until the final follow-up date or death from any cause, whichever occurred first. Follow-up was conducted through standardized outpatient evaluations, including physical examination, imaging, and laboratory testing, as well as structured telephone interviews for patients unable to attend in-person visits, with information obtained from patients or their immediate family members. Follow-up intervals were determined according to clinical guidelines: every 3 months during the first 2 years after enrollment, every 6 months from years 3 to 5, and annually thereafter unless clinical deterioration required more frequent monitoring. The last follow-up time was December 31, 2021. The median follow-up duration for the entire cohort was 20.75 months, during which 455 all-cause deaths occurred. The primary endpoint was OS, defined as the interval from baseline assessment to death from any cause or the last valid follow-up date. All follow-up data were independently reviewed by trained research staff to ensure accuracy and completeness.

### Definition of malnutrition

Malnutrition was assessed using the Patient-Generated Subjective Global Assessment (PG-SGA), a validated cancer-specific nutritional assessment tool that integrates patient-reported weight change, dietary intake, and symptoms with clinician-assessed muscle and fat status. PG-SGA scoring was completed jointly via patient self-report and professional clinician evaluation: patients independently finished the self-administered items regarding weight, appetite and gastrointestinal symptoms, while trained clinicians performed physical examination and scored the muscle/fat depletion component. Severe malnutrition was defined as a total PG-SGA score >8, indicating substantial nutritional depletion requiring prompt clinical intervention (19).

## Statistical analysis

Continuous variables were expressed as mean ± standard deviation or median (interquartile range), whereas categorical variables were presented as counts and percentages. Intergroup comparisons were performed using the *t*-test or Mann–Whitney *U* test for continuous variables and the *χ*^2^ test for categorical variables. The optimal cutoff value of IBI for predicting OS was determined by receiver operating characteristic (ROC) curve analysis using the maximum Youden index, with corresponding sensitivity and specificity calculated. Kaplan–Meier survival curves were generated to compare OS among different groups, and significance was assessed using the log-rank test. The survival percentages presented in curve comparisons denote the final cumulative survival probability at the end of the entire follow-up period, rather than OS at predefined fixed time points. Multivariate Cox proportional hazards regression models were constructed to evaluate the independent prognostic value of IBI, HGS, and the IBI-HGS grade. Three models were established: Model a (unadjusted), Model b (adjusted for age, sex, and TNM stage), and Model c (further adjusted for surgery, radiotherapy, chemotherapy, hypertension, diabetes, smoking, alcohol consumption, and family history of cancer). Restricted cubic spline (RCS) analyses were performed using Cox proportional hazards regression models to explore the potential nonlinear association between IBI and survival outcomes. Three knots were automatically selected according to the default settings of the rms package based on the distribution of IBI and HGS. The overall association and nonlinear component were assessed using the Wald test from the ANOVA of the fitted Cox proportional hazards regression models. Logistic regression analysis was performed to examine the association between the IBI-HGS grade and severe malnutrition, with odds ratios (ORs) and 95% confidence intervals (CIs) calculated. Sensitivity analyses were conducted after excluding patients who died within 3 months after baseline assessment while maintaining the same survival time origin to evaluate the robustness of the results. Statistical analyses were performed using IBM SPSS Statistics 26.0 and R software (version 4.3.2). All tests were two-sided, and a *p*-value < 0.05 was considered statistically significant.

## Results

### Comparison of inflammation-related indicators in predicting GC prognosis

A total of 16 common inflammation-related indicators were systematically compared to evaluate their prognostic performance in GC patients. As shown in Supplementary Table S1, predictive performance was assessed using the C-index and time-dependent ROC curve analyses. Among the evaluated indicators, the IBI showed potential prognostic value, with a C-index of 0.597 (95% CIs: 0.569–0.624). Time-dependent ROC curve analysis indicated that the IBI showed a relatively favorable discrimination ability for survival prediction (Supplementary Figure S1), although its predictive performance was not statistically significantly different from that of most conventional inflammatory markers (Table 1).

**Table 1:** Comparison the C-index of inflammation-related indicators in predicting the prognosis of patients with gastric cancer.

Inflammation-related indicators	C-index	C-index difference	*p* value
IBI	0.597 (0.569–0.624)	Ref	
CAR	0.589 (0.562–0.616)	−0.008 (−0.019 to 0.003)	0.178
NC	0.589 (0.561–0.617)	−0.007 (−0.017 to 0.002)	0.131
CALLY	0.589 (0.562–0.616)	−0.007 (−0.018 to 0.003)	0.169
LCR	0.586 (0.558–0.613)	−0.011 (−0.021 to 0.001)	0.054
PCS	0.579 (0.552–0.607)	−0.017 (−0.030 to 0.030)	0.010
NAR	0.578 (0.550–0.605)	−0.019 (−0.043 to 0.005)	0.123
NLR	0.577 (0.549–0.604)	−0.020 (−0.043 to 0.004)	0.094
SII	0.573 (0.546–0.601)	−0.023 (−0.047 to 0.001)	0.060
LCS	0.569 (0.545–0.594)	−0.027 (−0.050 to 0.005)	0.018
GPS	0.564 (0.539–0.588)	−0.033 (−0.053 to 0.012)	0.002
LA	0.564 (0.536–0.592)	−0.033 (−0.078 to 0.018)	0.185
NP	0.557 (0.529–0.585)	−0.039 (−0.068 to 0.011)	0.007
PAR	0.552 (0.523–0.580)	−0.045 (−0.082 to 0.008)	0.017
mGPS	0.544 (0.521–0.568)	−0.052 (−0.069 to 0.034)	<0.001
PLR	0.552 (0.524–0.579)	−0.045 (−0.091 to 0.011)	0.082

IBI, Inflammatory Burden Index; CAR, C-reactive protein-to-albumin ratio; NLR, Neutrophil-to-lymphocyte ratio; PLR, Platelet-to-lymphocyte ratio; SII, Systemic Immune-Inflammation Index; GPS, Glasgow Prognostic Score; mGPS, modified Glasgow Prognostic Score; LCR, Lymphocyte-to-C-reactive protein ratio; NAR, Neutrophil-to-albumin ratio; PAR, Platelet-to-albumin ratio; NC, Neutrophil-C-reactive protein score; LCS, Lymphocyte-C-reactive protein score; PCS, Platelet-C-reactive protein score; NP, Neutrophil–Platelet score; LA, Lymphocyte-Albumin score. Detailed calculation formulas for all biomarkers are provided in Supplementary Table S1.

### Development of the IBI-HGS grade

Based on ROC curve analysis, the optimal cutoff value of the IBI for predicting OS was identified as 7.83. Patients were accordingly stratified into low IBI (<7.83) and high IBI (≥7.83) groups. HGS was categorized using sex-specific cutoff values: low HGS was defined as <28 kg for males and <18 kg for females, whereas high HGS was defined as ≥ 28 kg for males or ≥ 18 kg for females. The IBI-HGS grade was subsequently established and classified into three risk groups: *low-risk grade* (low IBI + high HGS), *medium-risk grade* (low IBI + low HGS or high IBI + high HGS), and *high-risk grade* (high IBI + low HGS). Comparison of baseline characteristics showed that the high-risk group had a higher mean age (62.80 years) than the medium-risk (59.27 years) and low-risk (56.00 years) groups. A greater proportion of patients in the high-risk group presented with TNM stage III–IV disease. Laboratory assessments indicated that the high-risk group had elevated inflammatory markers, including white blood cell, neutrophil, platelet, and CRP levels, as well as impaired nutritional parameters, including lower red blood cell count, hemoglobin, and albumin levels. In addition, the high-risk group had higher PG-SGA scores, a greater prevalence of cancer cachexia, higher short-term and overall mortality rates, and an approximately 2-day longer hospital stay. All intergroup differences were statistically significant (Supplementary Table S2).

### Kaplan–Meier survival analysis

**Figure 1:**
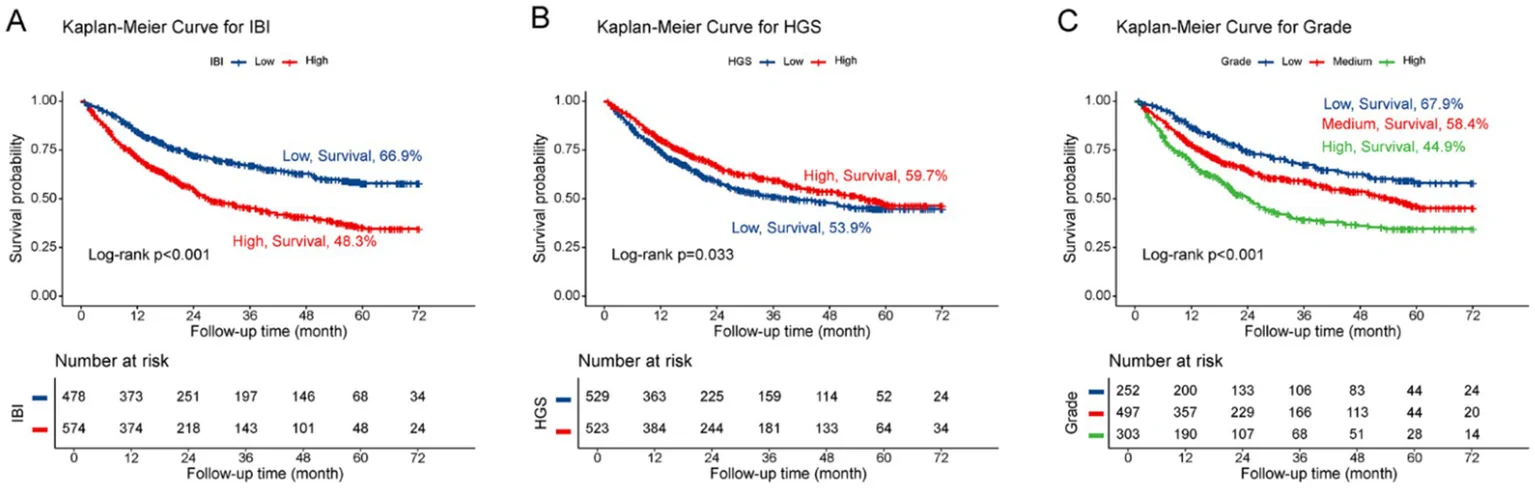
Kaplan–Meier curve of IBI, HGS, and IBI-HGS grade in patients with gastric cancer. **(A)** IBI; **(B)** HGS; **(C)** IBI-HGS grade. IBI, inflammatory burden index; HGS, handgrip strength.

Kaplan–Meier survival analysis showed that patients with high IBI had significantly worse OS than those with low IBI (48.3% vs. 66.9%, log-rank *p* < 0.001; Figure 1A). Similarly, patients with low HGS had poorer survival than those with high HGS (53.9% vs. 59.7%, log-rank *p* = 0.033; Figure 1B). Notably, the IBI-HGS grade provided robust prognostic stratification, with the high-risk group showing a markedly lower OS rate than the medium-risk and low-risk groups (44.9% vs. 58.4% vs. 67.9%, log-rank *p* < 0.001; Figure 1C). Subgroup analysis stratified by TNM stage further validated the prognostic value of the IBI-HGS grade across disease stages, particularly in advanced GC. Among patients with stage III disease, the low-risk group had a significantly higher OS rate than the medium-risk and high-risk groups (71.0% vs. 58.7% vs. 51.5%, *p* < 0.001). In stage IV patients, survival curves declined sharply within the first 24 months, with the high-risk group showing the steepest decline and persistent separation among the three risk groups (Supplementary Figure S3).

### Prognostic association of IBI-HGS grade with survival

**Figure 2:**
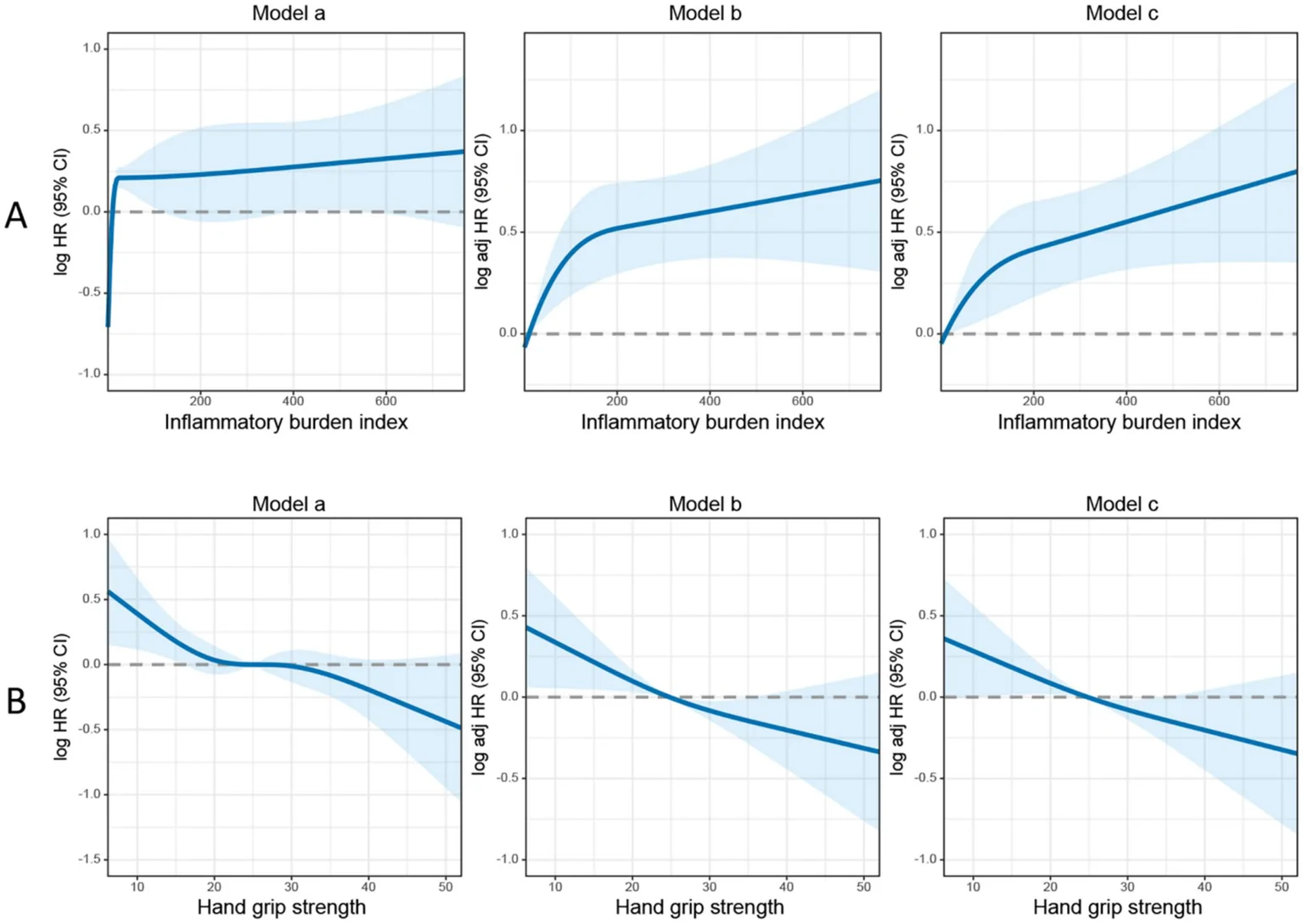
Association of IBI **(A)** and HGS **(B)** with all-cause mortality in patients with gastric cancer. **(A)** represents the dose–response relationship between IBI and log HR; **(B)** represents the dose–response relationship between HGS and log HR. Restricted cubic spline was adopted to evaluate nonlinear correlations between IBI/HGS and all-cause mortality. Three quantile-based knots were automatically assigned for each indicator. For IBI, knot locations were 1.179, 9.920, and 22.286, with the median IBI of 9.920 set as the reference value (log HR = 0). For HGS, knots were located at 13.6, 24.8, and 38.4, and the median HGS of 24.8 served as the reference point. Wald ANOVA tests were applied to assess nonlinear components; the *p* values for nonlinearity were 0.061 for IBI and 0.677 for HGS. IBI, Inflammatory Burden Index; HGS, handgrip strength. Model a: Unadjusted. Model b: Adjusted for age, sex, TNM stage. Model c: Adjusted for age, sex, TNM stage, surgery, radiotherapy, chemotherapy, hypertension, diabetes, smoking, drinking, family history.

RCS analysis visualized the trends linking IBI and HGS to all-cause mortality. Elevated IBI corresponded to higher mortality risk, while greater HGS was correlated with lower mortality risk (Figures 2A,B). Wald tests for nonlinearity returned *p* = 0.061 for IBI and *p* = 0.677 for HGS, indicating a marginally nonlinear tendency for IBI, whereas no statistically significant nonlinear association was detected for HGS (Figures 2A,B). Multivariate Cox proportional hazards regression models were further used to evaluate the prognostic impact of IBI, HGS, and the IBI-HGS grade. When analyzed as continuous, categorical, or quartile variables, both IBI and HGS independently predicted OS (Supplementary Tables S3, S4). For the IBI-HGS grade, unadjusted Model a showed that the medium-risk grade was associated with a 2.023-fold higher mortality risk (HR = 2.023, 95% CIs: 1.592–2.570, *p* < 0.001), whereas the high-risk grade was associated with a 3.165-fold increased risk (HR = 3.165, 95% CI: 2.246–4.129, *p* < 0.001). After adjustment in Model b, the HRs remained significant (medium-risk: HR = 1.765, 95% CIs: 1.387–2.246, *p* < 0.001; high-risk: HR = 2.318, 95% CIs: 1.765–3.044, *p* < 0.001). In the fully adjusted Model c, the medium-risk grade predicted a 69.1% higher mortality risk (HR = 1.691, 95% CIs: 1.323–2.162, *p* < 0.001), while the high-risk grade was associated with a 123.0% increased risk (HR = 2.230, 95% CI: 1.690–2.942, *p* < 0.001; Table 2). Sensitivity analysis excluding 56 patients who died within 3 months after diagnosis confirmed the robustness of these findings. After full adjustment, both the medium-risk (HR = 1.626, 95% CI: 1.258–2.101, *p* < 0.001) and high-risk (HR = 2.050, 95% CIs: 1.527–2.754, *p* < 0.001) grades remained independent predictors of poor OS (Supplementary Table S5).

**Table 2:** Multivariate Cox analysis was performed to evaluate the independent associations between the IBI-HGS grade and OS.

IBI-HGS Grade	Model a	*p* value	Model b	*p* value	Model c	*p* value
Normal	Ref		Ref		Ref	
Medium	2.023 (1.592, 2.570)	<0.001	1.765 (1.387, 2.246)	<0.001	1.691 (1.323, 2.162)	<0.001
High	3.165 (2.426, 4.129)	<0.001	2.318 (1.765, 3.044)	<0.001	2.230 (1.690, 2.942)	<0.001
*p* for trend		<0.001		<0.001		<0.001

IBI, Inflammatory Burden Index; HGS, handgrip strength.

Model a: Unadjusted.

Model b: Adjusted for age, sex, TNM stage.

Model c: Adjusted for age, sex, TNM stage, surgery, radiotherapy, chemotherapy, hypertension, diabetes, smoking, drinking, family history.

### Association between IBI-HGS grade and severe malnutrition

Among the GC patients, 515 (48.7%) had severe malnutrition at baseline. Stepwise elevated median PG-SGA scores were observed across the three risk strata: low-risk group 5.00 (IQR: 3.00–9.00), medium-risk group 7.00 (IQR: 4.00–11.00), and high-risk group 9.00 (IQR: 6.00–12.00). Post-hoc pairwise intergroup comparisons demonstrated statistically significant differences between all paired subgroups: low-risk versus medium-risk (*p* < 0.001), low-risk versus high-risk (*p* < 0.001), and medium-risk versus high-risk (*p* < 0.001), which clearly reflected the graded disparity in nutritional depletion across risk tiers (Supplementary Table S2). Logistic regression analysis demonstrated a stepwise association between increasing IBI-HGS grade and higher odds of baseline severe malnutrition. In unadjusted Model a, the medium-risk grade was associated with higher odds of baseline severe malnutrition (OR = 1.655, 95% CIS: 1.247–2.198, *p* < 0.001), whereas the high-risk grade showed a stronger association with baseline severe malnutrition (OR = 3.355, 95% CIs: 2.350–4.791, *p* < 0.001). After partial adjustment in Model b, these associations remained significant (medium-risk: OR = 1.573, 95% CIs: 1.179–2.098, *p* = 0.002; high-risk: OR = 3.047, 95% CIs: 2.111–4.398, *p* < 0.001). In the fully adjusted Model c, the medium-risk grade was associated with a 56.2% higher odds of baseline severe malnutrition (OR = 1.562, 95% CIs: 1.167–2.091, *p* = 0.003), whereas the high-risk grade was associated with a 205.8% higher odds of baseline severe malnutrition (OR = 3.058, 95% CIs: 2.110–4.431, *p* < 0.001) (Table 3).

**Table 3:** Logistic regression analysis was used to explore the relationship between IBI-HGS grade and severe malnutrition (PGSGA>8).

IBI-HGS grade	Model a	*p* value	Model b	*p* value	Model c	*p* value
Normal	Ref		Ref		Ref	
Medium	1.655 (1.247, 2.198)	<0.001	1.573 (1.179, 2.098)	0.002	1.562 (1.167, 2.091)	0.003
High	3.355 (2.350, 4.791)	<0.001	3.047 (2.111, 4.398)	<0.001	3.058 (2.110, 4.431)	<0.001
*p* for trend		<0.001		<0.001		<0.001

IBI, Inflammatory Burden Index; HGS, handgrip strength; PG-SGA, Patient-Generated Subjective Global Assessment.

Model a: Unadjusted.

Model b: Adjusted for age, sex, TNM stage.

Model c: Adjusted for age, sex, TNM stage, surgery, radiotherapy, chemotherapy, hypertension, diabetes, smoking, drinking, family history.

## Discussion

This multicenter cohort study developed a novel prognostic grading system combining the IBI and HGS, termed the IBI-HGS grade, and evaluated its independent associations with OS and baseline severe malnutrition in GC patients. The findings demonstrated that the IBI-HGS grade, as a dual-dimensional indicator integrating systemic inflammatory burden and skeletal muscle function, outperformed individual inflammatory or nutritional markers in prognostic stratification. A high-risk IBI-HGS grade was associated with poorer overall survival and higher prevalence of severe malnutrition at baseline, providing a practical tool for risk assessment and clinical decision-making in GC patients.

Systemic inflammation is a hallmark of cancer progression and a major contributor to poor prognosis in GC. Elevated systemic inflammation, characterized by increased CRP levels and dysregulated immune cell ratios, promotes tumor proliferation, invasion, and immunosuppression while exacerbating nutritional depletion and muscle wasting (12, 20, 21). Among the various inflammation-related indicators evaluated, the present study demonstrated that the IBI—an integrated index incorporating CRP, neutrophil count, and lymphocyte count—showed potential prognostic value for GC survival. Compared with individual inflammatory indicators, the IBI may provide additional prognostic information by integrating multiple aspects of systemic inflammatory status. This may be attributed to its ability to capture the overall inflammatory burden, whereas individual indicators such as NLR, PLR, or CRP reflect only specific components of the inflammatory response. Consistent with previous studies, elevated IBI was associated with worse OS in GC, supporting its role as a robust inflammatory prognostic biomarker (11). However, the IBI reflects only inflammation and does not account for muscle function or nutritional status, which are critical determinants of host resilience.

HGS, a simple and objective measure of skeletal muscle mass and function, is a well-established marker of sarcopenia and malnutrition in cancer patients (17, 22, 23). Reduced HGS reflects impaired physical reserve, poor treatment tolerance, and increased mortality in GC (18, 24). Although the present study confirmed that low HGS was associated with inferior survival, its prognostic performance as an individual indicator was limited (25). This finding is consistent with previous studies showing that HGS alone has limited predictive value in gastrointestinal cancer cohorts because it does not account for systemic inflammation, a major driver of muscle wasting. The complementary roles of inflammation and muscle function motivated the integration of IBI and HGS to overcome the limitations of single-dimensional assessments.

To our knowledge, this study is the first to establish an IBI-HGS grading system for GC. By stratifying patients into low-, medium-, and high-risk groups according to combined inflammatory and muscle function status, the IBI-HGS grade provided more refined prognostic stratification than either indicator alone. High-risk patients (high IBI + low HGS) had substantially elevated risks of both mortality and severe malnutrition, even after adjustment for major confounding factors. Mechanistically, inflammation and muscle wasting form a vicious cycle in GC: proinflammatory cytokines promote muscle protein degradation, whereas sarcopenia further impairs immune function and aggravates inflammation (26, 27). The IBI-HGS grade captures this bidirectional interaction and provides a comprehensive assessment of host pathophysiological status.

The IBI-HGS grade has important clinical implications. First, it provides a simple and cost-effective prognostic tool. Both the IBI, derived from routine blood tests, and HGS, measured at the bedside, are readily accessible in clinical practice, eliminating the need for complex imaging or time-consuming nutritional assessments. Second, it enables precise risk stratification in GC patients, particularly those with advanced disease. Subgroup analysis confirmed that the IBI-HGS grade effectively stratified survival outcomes in stage III–IV GC, a population with highly heterogeneous prognosis. Third, it identifies patients at high risk of severe malnutrition, allowing timely targeted interventions, such as nutritional support, anti-inflammatory treatment, and exercise training, to improve physical reserve and prognosis.

However, several limitations should be acknowledged. First, this was an observational study, and residual confounding factors, such as specific nutritional interventions and detailed medication history, could not be completely excluded. Second, the study cohort consisted exclusively of Chinese patients; given that genetic, lifestyle, and body composition factors vary across ethnicities and may influence inflammation-muscle interactions, our findings require validation in multi-ethnic cohorts. Third, only baseline IBI and HGS were analyzed, and dynamic changes in these indicators during treatment were not evaluated; consequently, their predictive value for treatment response remains unclear; longitudinal studies are needed to clarify whether serial IBI-HGS monitoring can predict clinical outcomes and nutritional decline. We further hypothesize that continuous serial reassessment of the IBI-HGS grade throughout anti-tumor treatment may deliver unique real-time predictive value. Fluctuations in the combined inflammatory burden and skeletal muscle function reflected by serial IBI-HGS measurements have the potential to early identify patients prone to poor treatment response or progressive, irreversible nutritional deterioration. Longitudinal prospective studies are required to verify the predictive performance of dynamic IBI-HGS surveillance, which may enable clinicians to adjust nutritional support and anti-inflammatory interventions in a timely manner during the course of antitumor therapy. Fourth, this study focused on GC, and the applicability of the IBI-HGS grade to other cancer types requires further investigation. Fifth, although patients were recruited from multiple centers in this study, rigorous cross-center validation was not conducted. Future prospective multicenter studies with independent validation cohorts are needed to further verify the generalizability of our findings. Sixth, because PG-SGA, IBI, and HGS were assessed concurrently at baseline, our findings should be interpreted as associations with baseline severe malnutrition rather than prediction of incident malnutrition. Moreover, although HGS provides an objective measure of muscle function, some overlap may exist between HGS and the functional component of PG-SGA. Future prospective studies with longitudinal nutritional assessments are warranted to further evaluate the predictive value of these indicators for subsequent nutritional deterioration.

## Conclusion

This multicenter study demonstrated that the IBI-HGS grade, integrating inflammation and muscle function, independently predicts OS and severe malnutrition in GC. This practical biomarker enables robust prognostic stratification and supports personalized clinical management.

## Data Availability

The original contributions presented in the study are included in the article/Supplementary material, further inquiries can be directed to the corresponding author/s.
