# Supplementary material for: Combined Inflammatory Burden Index and hand grip strength for prognostic assessment in gastric cancer patients: a multicenter cohort study
Source: Front Nutr. 2026 Sep 9;13:1906113. doi: 10.3389/fnut.2026.1906113 (PMC13597362; doi:10.3389/fnut.2026.1906113)
Supplement: Supplementary file 1 [file Supplementary_file_1.docx]

**Figure S1.** Comparison the effectiveness of inflammation-related indicators in predicting the prognosis of patients with gastric cancer.

**Notes:** IBI, Inflammatory Burden Index; CAR, C-reactive protein-to-albumin ratio; NLR, Neutrophil-to-lymphocyte ratio; PLR, Platelet-to-lymphocyte ratio; SII, Systemic Immune-Inflammation Index; GPS, Glasgow Prognostic Score; mGPS, modified Glasgow Prognostic Score; LCR, Lymphocyte-to-C-reactive protein ratio; NAR, Neutrophil-to-albumin ratio; PAR, Platelet-to-albumin ratio; NC, Neutrophil-C-reactive protein score; LCS, Lymphocyte-C-reactive protein score; PCS, Platelet-C-reactive protein score; NP, Neutrophil-Platelet score; LA, Lymphocyte-Albumin score.

**Figure S2.** The Cut-off of IBI in patients with gastric cancer.

**Notes:** IBI, Inflammatory Burden Index.

**Figure S3.** Subgroup survival analysis of IBI-HGS grade based on pathological stage.

**Notes：**A, Stage I; B, Stage II; C, Stage III; D, Stage IV. IBI, Inflammatory Burden Index; HGS, handgrip strength.

**Table S1.** The inflammatory biomarkers evaluated in this study.

| Biomarker name | Biomarker formulas |
| --- | --- |
| C-reactive protein-to-albumin ratio (CAR) | C-reactive protein (mg/L)/albumin (g/L) |
| Platelet-to-albumin ratio (PAR) | Platelet (/uL)/albumin (g/L) |
| Neutrophil-to-albumin ratio (NAR) | Neutrophil (/uL)/albumin (g/L) |
| Lymphocyte-to-C-reactive protein ratio (LCR) | Lymphocyte (/uL)/C-reactive protein (mg/L) |
| Platelet-to-Lymphocyte ratio (PLR) | Platelet (/uL)/lymphocyte (/uL) |
| Neutrophil-to-Lymphocyte ratio (NLR) | Neutrophil (/uL)/lymphocyte (/uL) |
| Systemic-Immune-Inflammation Index (SII) | Platelet (/uL)×Neutrophil (/uL)/lymphocyte (/uL) |
| C-reactive protein-Neutrophil-Lymphocyte ratio (Inflammatory burden index, IBI) | C-reactive protein (mg/L)×Neutrophil (/uL)/lymphocyte (/uL) |
| Glasgow Prognostic Score (GPS) | C-reactive protein≤10mg/L and albumin ≥35g/L: 0 score  C-reactive protein≤10mg/L or albumin < 35g/L: 1 score  C-reactive protein >10mg/L and albumin < 35g/L: 2 score |
| Modified Glasgow Prognostic Score (mGPS) | C-reactive protein≤10mg/L and albumin ≥35g/L: 0 score  C-reactive protein≤10mg/L and albumin < 35g/L: 0 score  C-reactive protein > 10mg/L: 1 score  C-reactive protein >10mg/L and albumin < 35g/L: 2 score |
| Lymphocyte C-reactive protein score (LCS) | Lymphocyte ≥1×10^9/L and C-reactive protein ≤ 3 mg/L: 0 score;  Lymphocyte ≤1×10^9/L or C-reactive protein ≥ 3 mg/L: 1 score;  Lymphocyte ≤1×10^9/L and C-reactive protein ≥ 3 mg/L: 2 score |
| Neutrophil-C-reactive protein score(NC) | Neutrophil (/uL) × C-reactive protein score (mg/L) |
| Platelet-C-reactive protein score (PCS) | Platelet (/uL) × C-reactive protein score (mg/L) |
| Neutrophil-Platelet score (NP) | Neutrophil (/uL) × Platelet score (/uL) |
| Lymphocyte-Albumin score (LA) | Lymphocyte (/uL) × Albumin (g/dL) score |

**Table S2.** Characteristics were stratified by IBI-HGS grade in patients with gastric cancer.

| **Characteristic** | Grade | | | |
| --- | --- | --- | --- | --- |
|  | Low (n=252) | Medium (n=497) | High (n=303) | p value |
| Sex, male, n (%) | 179 (71.0) | 348 (71.2) | 211 (69.6) | 0.855 |
| Age, years, mean (SD) | 56.00 (11.22) | 59.27 (11.29) | 62.80 (11.87) | <0.001 |
| Hypertension, yes, n (%) | 27 (10.7) | 92 (18.5) | 59 (19.5) | 0.01 |
| Diabetes, yes, n (%) | 15 ( 6.0) | 46 (9.3) | 37 (12.2) | 0.041 |
| Smoking yes, n (%) | 110 (43.7) | 238 (47.9) | 144 (47.5) | 0.521 |
| Drinking, yes, n (%) | 53 (21.0) | 134 (27.0) | 68 (22.4) | 0.139 |
| Family history, yes, n (%) | 37 (14.7) | 100 (20.1) | 33 (10.9) | 0.002 |
| TNM stage, n (%) |  |  |  |  |
| Stage I | 27 (10.7) | 52 (10.5) | 25 ( 8.3) | 0.014 |
| Stage II | 68 (27.0) | 97 (19.5) | 58 (19.1) |  |
| Stage III | 93 (36.9) | 179 (36.0) | 99 (32.7) |  |
| Stage IV | 64 (25.4) | 169 (34.0) | 121 (39.9) |  |
| Surgery, yes, n (%) | 215 (85.3) | 383 (77.1) | 217 (71.6) | 0.001 |
| Radiotherapy, yes, n (%) | 6 ( 2.4) | 10 ( 2.0) | 8 ( 2.6) | 0.84 |
| Chemotherapy, yes, n (%) | 161 (63.9) | 302 (60.8) | 161 (53.1) | 0.025 |
| IBI (median (IQR)) | 3.22 (1.28, 5.11) | 9.92 (3.26, 43.28) | 52.67 (18.05, 192.13) | <0.001 |
| HGS (median (IQR)) | 32.20 (28.20, 38.30) | 25.60 (19.10, 33.20) | 17.00 (13.70, 22.25) | <0.001 |
| White blood cells (median (IQR)) | 5.05 (4.12, 6.00) | 5.90 (4.50, 7.82) | 6.72 (5.23, 8.68) | <0.001 |
| Neutrophil (median (IQR)) | 2.42 (1.80, 3.30) | 3.38 (2.26, 5.25) | 4.66 (3.20, 6.16) | <0.001 |
| Lymphocyte (median (IQR)) | 1.60 (1.19, 1.92) | 1.40 (0.99, 1.85) | 1.20 (0.92, 1.60) | <0.001 |
| Platelets (median (IQR)) | 208.50 (158.75, 264.25) | 217.00 (165.00, 275.00) | 231.00 (170.00, 288.50) | 0.07 |
| Red blood cells (median (IQR)) | 4.31 (3.86, 4.74) | 4.11 (3.60, 4.56) | 3.90 (3.50, 4.43) | <0.001 |
| Hemoglobin (median (IQR)) | 125.00 (108.75, 140.00) | 121.00 (101.00, 135.00) | 113.00 (94.00, 129.00) | <0.001 |
| Albumin (median (IQR)) | 41.00 (38.58, 43.62) | 38.90 (35.30, 42.50) | 35.90 (31.95, 39.60) | <0.001 |
| C-reactive protein (median (IQR)) | 2.00 (0.80, 3.10) | 3.50 (2.12, 12.00) | 15.90 (6.00, 42.15) | <0.001 |
| PGSGA score (median (IQR)) | 5.50 (3.00, 9.00) | 7.00 (4.00, 11.00) | 9.00 (6.00, 12.00) | <0.001 |
| Cachexia, yes, n (%) | 117 (46.4) | 244 (49.1) | 166 (54.8) | 0.121 |
| 90-day outcome | 5 ( 2.0) | 26 ( 5.2) | 25 ( 8.3) | 0.005 |
| Global quality of life score (median (IQR)) | 45.00 (42.00, 50.50) | 48.00 (43.00, 56.00) | 50.00 (44.75, 60.00) | <0.001 |
| Status, death, n (%) | 81 (32.1) | 207 (41.6) | 167 (55.1) | <0.001 |
| Length of hospitalization (median (IQR)) | 9.00 (6.00, 13.00) | 9.00 (6.00, 14.00) | 11.00 (8.00, 15.00) | <0.001 |
| Hospitalization expenses(median (IQR)) | 19274.85 (11267.60, 56137.00) | 20542.50 (11167.20, 56316.00) | 24195.60 (12815.25, 57321.45) | 0.312 |

**Notes:** IBI, Inflammatory Burden Index; HGS, handgrip strength; PG-SGA, Patient-Generated Subjective Global Assessment.

**Table S3.** Association between IBI and survival of patients with gastric cancer.

| IBI | Model a | p value | Model b | p value | Model c | p value |
| --- | --- | --- | --- | --- | --- | --- |
| Continuous (per SD) | 1.135 (1.038, 1.241) | 0.005 | 1.194 (1.093, 1.305) | <0.001 | 1.195 (1.092, 1.307) | <0.001 |
| Cutoff value |  |  |  |  |  |  |
| C1 (<7.83) | ref |  | ref |  | ref |  |
| C2 (≥7.83） | 1.954 (1.610, 2.372) | <0.001 | 1.603 (1.317, 1.951) | <0.001 | 1.505 (1.225, 1.847) | <0.001 |
| Quartiles |  |  |  |  |  |  |
| Q1 (<3.47) | ref |  | ref |  | ref |  |
| Q2 (3.47-9.92) | 1.355 (1.014, 1.811) | 0.040 | 1.158 (0.864, 1.552) | 0.325 | 1.129 (0.841, 1.516) | 0.418 |
| Q3 (9.92-52.44) | 1.906 (1.450, 2.506) | <0.001 | 1.421 (1.076, 1.878) | 0.013 | 1.323 (0.994, 1.762) | 0.055 |
| Q4 (≥52.44) | 2.234 (1.713, 2.914) | <0.001 | 1.869 (1.430, 2.444) | <0.001 | 1.683 (1.275, 2.222) | <0.001 |
| P for trend |  | <0.001 |  | <0.001 |  | <0.001 |

Notes: IBI, Inflammatory Burden Index.

Model a: Unadjusted.

Model b: Adjusted for age, sex, TNM stage.

Model c: Adjusted for age, sex, TNM stage, surgery, radiotherapy, chemotherapy, hypertension, diabetes, smoking, drinking, family history.

**Table S4.** Association between HGS and survival of patients with gastric cancer.

| HGS | Model a | p value | Model b | p value | Model c | p value |
| --- | --- | --- | --- | --- | --- | --- |
| Continuous (per SD) | 0.839 (0.776,0.907) | <0.001 | 0.901 (0.830,0.979) | 0.014 | 0.903 (0.831,0.980) | 0.015 |
| Cutoff value |  |  |  |  |  |  |
| C1 (Men<28 or Women<18 ) | ref |  | ref |  | ref |  |
| C2 (Men≥28 or Women≥18 ） | 0.569 (0.472,0.685) | <0.001 | 0.661 (0.547,0.800) | <0.001 | 0.651 (0.537,0.789) | <0.001 |
| Quartiles |  |  |  |  |  |  |
| Q1 (<17.5) | ref |  | ref |  | ref |  |
| Q2 (17.5-24.8) | 0.812 (0.633,1.040) | 0.099 | 0.871 (0.680,1.117) | 0.277 | 0.842 (0.653,1.084) | 0.182 |
| Q3 (24.8-32.3) | 0.640 (0.501,0.816) | <0.001 | 0.758 (0.592,0.970) | 0.028 | 0.743 (0.579,0.955) | 0.020 |
| Q4 (≥32.3) | 0.477 (0.362,0.628) | <0.001 | 0.617 (0.466,0.817) | 0.001 | 0.598 (0.449,0.796) | <0.001 |
| P for trend |  | <0.001 |  | <0.001 |  | <0.001 |

Notes: HGS, handgrip strength

Model a: Unadjusted.

Model b: Adjusted for age, sex, TNM stage.

Model c: Adjusted for age, sex, TNM stage, surgery, radiotherapy, chemotherapy, hypertension, diabetes, smoking, drinking, family history.

**Table S5.** Sensitivity analysis was conducted by excluding mortalities within the first 3 months (56 patients).

| IBI-HGS Grade | Model a | p value | Model b | p value | Model c | p value |
| --- | --- | --- | --- | --- | --- | --- |
| Normal | Ref |  | Ref |  | Ref |  |
| Medium | 1.952 (1.521,2.505) | <0.001 | 1.714 (1.334,2.204) | <0.001 | 1.626 (1.258,2.101) | <0.001 |
| High | 2.926 (2.206,3.881) | <0.001 | 2.162 (1.618,2.888) | <0.001 | 2.050 (1.527,2.754) | <0.001 |
| P for trend |  | <0.001 |  | <0.001 |  | <0.001 |

Notes: IBI, Inflammatory Burden Index; HGS, handgrip strength.

Model a: Unadjusted.

Model b: Adjusted for age, sex, TNM stage.

Model c: Adjusted for age, sex, TNM stage, surgery, radiotherapy, chemotherapy, hypertension, diabetes, smoking, drinking, family history.
